# CD44^+^ cells enhance pro-tumor stroma in the spatial landscape of colorectal cancer leading edge

**DOI:** 10.1038/s41416-025-02968-9

**Published:** 2025-03-12

**Authors:** Feiyu Tang, Yongwei Zhu, Jia Shen, Bowen Yuan, Xiang He, Yuxi Tian, Liang Weng, Lunquan Sun

**Affiliations:** 1 Xiangya Cancer Center, Xiangya Hospital, Central South University, Changsha, China; 2 Key Laboratory of Molecular Radiation Oncology Hunan Province, Changsha, China; 3 Center for Biotherapy, Sun Yat-Sen Memorial Hospital, Sun Yat-Sen University, Guangzhou, China; 4 Department of Neurosurgery, Xiangya Hospital, Central South University, Changsha, China; 5 Hunan International Scientific and Technological Cooperation Base of Brain Tumor Research, Xiangya Hospital, Central South University, Changsha, China; 6 Department of Pathology, Third Xiangya Hospital, Central South University, Changsha, China; 7 Department of Geriatric Respiratory and Critical Care Medicine, Xiangya Hospital, Central South University, Changsha, China; 8 Department of Pathology, School of Basic Medical Sciences, Peking University Third Hospital, Peking University Health Science Center, Beijing, China; 9 FuRong Laboratory, Changsha, China

**Keywords:** Computational biology and bioinformatics, Cancer microenvironment

## Abstract

**Background:**

The heterogeneity of tumors significantly impacts on colorectal cancer (CRC) progression. However, the influence of this heterogeneity on the spatial architecture of CRC remains largely unknown.

**Methods:**

Spatial transcriptomic (ST) analysis of AOM/DSS-induced colorectal cancer (CRC), integrated with single-cell RNA sequencing, generated a comprehensive spatial atlas of CRC. Pseudotime trajectory, stemness evaluation, and cell-cell communication analyses explored how CD44^+^ tumor cells at the leading edge remodel the tumor microenvironment (TME). In vitro experiments and immunofluorescence staining of clinical samples validated pleiotrophin (PTN) signaling in promoting cancer-associated fibroblasts (CAFs) phenotypic transition and CRC progression.

**Results:**

Our findings revealed a distinctive layered ring-like structure within CRC tissues, where CD44^+^ tumor cells exhibiting high stemness were positioned at the tumor’s leading edge. Inflammatory CAFs (iCAFs)-like, myofibroblastic CAFs (myCAFs)-like cells and pro-tumorigenic neutrophils primarily located at the tumor edge, in proximity to CD44^+^ tumor cells. CD44^+^ tumor cells then triggered the phenotypic transition of CAFs into iCAF-like and myCAF-like cells through PTN signaling.

**Conclusions:**

Our results provide distinctive insights into how tumor heterogeneity reshapes the TME at the leading edge of tumor, thereby promoting CRC progression.

## Introduction

Colorectal cancer (CRC) is the third most common malignancy worldwide, with 5-year and 10-year survival rates of 65% and 58%, respectively [1]. Despite clinical trials showing that targeted therapy or immunotherapy can improve survival in advanced CRC, treatment options remain limited [2]. Thus, uncovering the underlying mechanism for cancer progression is urgently needed. The tumor microenvironment (TME) profoundly impacts local and systemic immune responses, as well as tumor formation, progression, and treatment outcomes. The complex interactions between tumor cells and the surrounding TME are critical determinants of tumor progression [3]. Hence, further exploration of the crosstalk between tumor cells and TME holds promise for elucidating the mechanisms of tumor progression and discovering novel therapeutic targets.

Tumor heterogeneity plays a pivotal role in tumorigenesis, tumor progression, and therapeutic responses. Both tumor cells and components of the TME exhibit diverse spatial characteristics. Gene expression alterations occur during tumor progression to adapt to the conditions necessary for tumor expansion, with the coexistence of subpopulations harboring distinct gene mutations or expression profiles, which contribute to the generation and maintenance of tumor heterogeneity. For instance, various cancer stem cell (CSC) subtypes have been identified with distinct distributions and functions within tumors. Some are located at the leading edge of tumor, potentially contributing to metastasis, while others reside within the tumor, possibly influencing therapy resistance [4]. Recently, utilization of single-cell sequencing (scRNA-seq) has deepened our understanding of tumor heterogeneity across different components in the TME, including various immune cells and stromal cells. For example, the cancer-associated fibroblasts (CAFs) are found to consist of two major subtypes, myofibroblastic CAFs (myCAFs) and inflammatory CAFs (iCAFs), which significantly influence cellular growth, proliferation and pro-metastatic programs [5]. Additionally, the terms N1 and N2 have been employed to describe the antitumor and pro-tumor phenotypes of tumor-associated neutrophils (TANs), respectively [6]. Nevertheless, the spatial features of the heterogeneity in colorectal cancer tissues remain to be resolved, and the mechanisms governing the diversity between these distinct cell subtypes are not well defined.

In addition to the spatial heterogeneity within tumors, the intricate interplay among cells holds a pivotal role in sustaining tumor heterogeneity and progression. Numerous studies have indicated that CSCs possess the capability to reshape the TME, supported by the evidence illustrating the encapsulation of CSCs within an immunosuppressive TME context [7]. For example, CSCs orchestrate the recruitment of myeloid-derived suppressor cells (MDSCs) and regulatory T cells (Tregs) by expressing TGF-β. Moreover, they induce the conversion of vasculature-derived monocytes into tumor associated macrophages (TAMs) by expressing periostin [8]. Notably, some reports depict the TGF-β-induced transformation of CSCs into CAFs to mimic the formation of CSC niche [9]. Reciprocally, the TME can profoundly influences the maintenance of tumor cell stemness. However, the distribution of heterogeneity among CSCs in CRC and the mechanisms by which CSCs influence the reshaping of the TME to promote tumor progression remain unclear.

In this study, we applied spatial transcriptomics (ST) on six samples using a widely employed AOM/DSS mouse model, a chemically induced animal model that sequentially administers the tumor-initiating agent azoxymethane and the inflammation-inducing agent dextran sodium sulfate (hereafter referred to as AOM/DSS). In addition, we integrated previously published scRNA-seq data to thoroughly investigate the spatial heterogeneity of the TME in CRC. Our study further examined the crosstalk between CSC-like cells and infiltrating cells, specifically distributed at the tumor leading edge, shedding light on their impact on the CRC ecosystem.

## Materials and methods

### AOM/DSS mouse model construction

Female C57BL/6 mice, aged 7-8 weeks, were procured from Hunan SJA Laboratory Animal Co., LTD and subjected to mating. The construction of the mouse model for AOM/DSS-induced colorectal cancer followed previously described protocols [10]. In brief, on day 0, mice received a single peritoneal injection of AOM (10 mg/ml) solution; on day 7, mice were treated with 2.5% DSS solution for a week, followed by free water for two weeks. This cycle was repeated twice. Disease progression was determined through body weight changes and the presence of rectal bleeding. The procedure concluded at day 80. The colorectal region, extending from the cecum to the anus, was dissected, rinsed twice with saline solution, cut open longitudinally, and then rolled up using the “Swiss-roll” technique before being fixed and embedded in paraffin [11].

A total of four mice were used for AOM/DSS-induced CRC modeling, while two untreated mice served as the control group, resulting in a total of six experimental mice. Group assignment was conducted in a double-blinded manner.

All animal studies were approved by the Institutional Animal Care and Use Committee (IACUC) of Central South University (IRB approval #2022020297). Mice were housed in specific pathogen-free facilities under a 12-hour light/dark cycle and controlled temperature (20–25 °C).

### Clinical CRC samples preparation

The CRC tissues used in this study were all surgical samples obtained from clinical patients. None of the patients had received any neoadjuvant therapy following their CRC diagnosis. Detailed patient information, including tumor location and size, can be found in the supplementary information.

For subsequent immunohistochemistry (IHC) and multi-color immunofluorescence staining assays, the surgically resected CRC tissues were washed twice with pre-cold PBS buffer containing 5% bovine serum albumin and 1% penicillin/streptomycin, and fixed in 4% paraformaldehyde for 48 hours. Dehydration and embedding in paraffin was performed following routine methods. The paraffin blocks were cut into 4 μm slides and adhered on the slides glass.

For subsequent in vitro experiments involving cancer-associated fibroblasts (CAFs) isolated from CRC tissues, the surgically resected CRC tissues were immediately submerged in PBS buffer containing 5% bovine serum albumin and 1% penicillin/streptomycin and sent to the laboratory for processing as soon as possible. Then, tissues were rinsed three times with pre-cold PBS buffer containing 1% penicillin/streptomycin, and cut into 1~2 mm-diameter pieces.

For additional information, see Supplemental Materials.

## Results

### Revelation of CRC unique architectures spatial transcriptomics

**Fig. 1:**
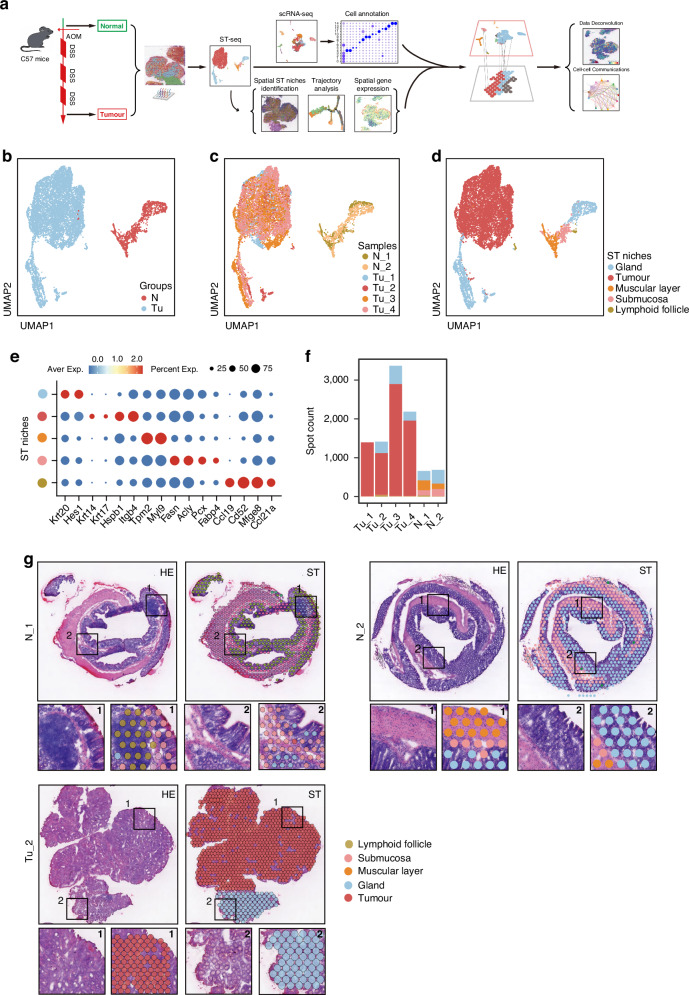
Landscape of CRC with ST. **a** Workflow depicting the construction of the AOM/DSS-induced CRC mouse model and sample processing for ST. **b** Uniform Manifold Approximation and Projection (UMAP) visualization of the dimensional reduction results for gene expression data from all ST spots in the Control and Tumor groups. Each spot on the UMAP plot represents the complete transcriptomic profile within a single ST spot. **c** UMAP visualization of the dimensional reduction results separately for each of the six mouse samples. **d** UMAP visualization of the dimensional reduction results for five intestinal tissue categories based on transcriptomic profiles. **e** Biomarkers used for annotation of tissue niches of ST-seq. **f** Spot counts for each sample derived from ST-seq. **g** Upper left panel, H&E staining of representative sections N_1, N_2 and Tu_2; upper right panel, distribution of tissue types for representative sections N_1, N_2 and Tu_2, respectively; lower panel, zoom-in field annotated with specific ST niches. Other sections can be seen in Supplementary Fig. S1.

To comprehensively analyze the spatial heterogeneity of CRC, we collected 6 tissue specimens from wildtype and AOM/DSS mouse model (Fig. 1a). Two of these specimens were colon tissues from control mice without AOM/DSS induction (N_1 and N_2), and four were tumor tissues from mice with AOM/DSS induction, primarily from the distal colons (Tu_1~Tu_4). Then ST sequencing was conducted by 10× Genomics Visium Platform. After filtering out non-coding RNAs (ncRNA) and mitochondrial-related protein-coding genes, we obtained valid sequencing data from 9711 loci, and the average sequencing depth per locus was 22,296 UMIs and 5358 genes (Supplementary Table S1). Subsequently, we performed a population analysis using UMAP (Uniform Manifold Approximation and Projection), which revealed that loci from normal and tumor stages exhibited completely different gene expression profiles (spots closer on the UMAP plot indicate greater transcriptomic similarity, whereas those farther apart reflect greater differences) (Fig. 1b, c). Based on transcriptomic features and H&E histological morphology, we identified and annotated five tissue populations: normal glandular tissues (annotated by Krt20 and Hes1), tumor tissues (annotated by Krt14, Krt17, Hspb1 and Itgb4), muscularis tissues (annotated by Tpm2 and Myl9), submucosal tissues (annotated by Fasn, Acly, Pcx and Fabp4) and lymphoid follicular structures (annotated by Mfge8 and Ccl21a) (Fig. 1d–f; Supplementary Fig. S1a). Due to the limited resolution of ST where each individual ST spot featuring a 50 μm diameter encompassed multiple cells (typically ranging from 1 to 10 cells) with mixed mRNA information that rendered cell annotation not precisely, we therefore performed histological H&E staining to further characterize the cell populations in different sections of each mouse (Fig. 1g; Supplementary Fig. S1b). Combined the ST maps with H&E images, the tissue populations were identified as normal gland compartments, tumor compartment, muscular compartment, submucosa compartment and lymphoid follicle compartment, with varying proportions in different tissues (Fig. 1f; Supplementary Fig. S1c). Those observations present a unique spatial organization of CRC.

### Spatial features of colorectal epithelium niches

**Fig. 2:**
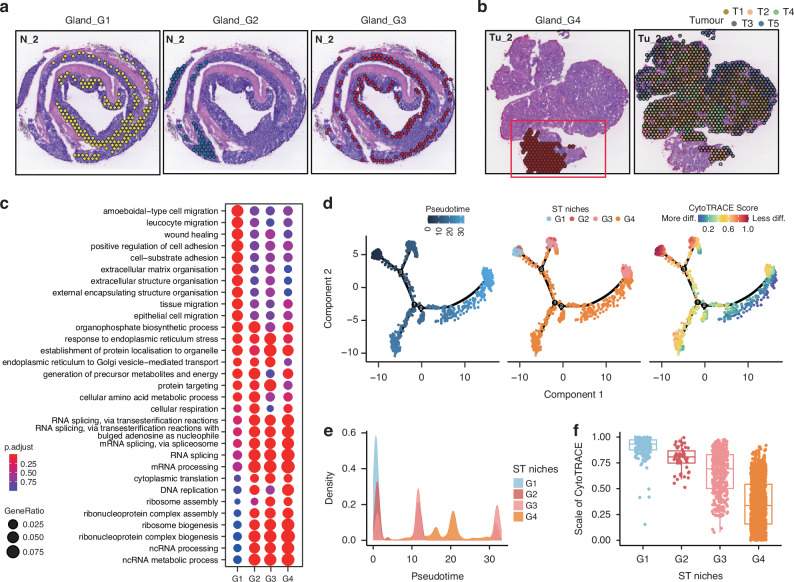
Progression of colorectal epithelium. **a** Distribution of G1 (left panel), G2 (middle panel), and G3 (right panel) niches for section N_2, respectively. **b** Distribution of G4 (left panel), and T1–T5 (right panel) niches for section Tu_2, respectively. **c** Functional enrichment analysis (Gene Ontology, GO) defining spatial G1–G4 niches, presented as a dot plot. **d** Pseudotime trajectory analysis showing glandular epithelium evolution ordered in a branched pseudotime trajectory. Spatial niches are colored by pseudotime (left panel), niche type (middle panel), and CytoTRACE score (right panel). **e** Distributions of gland-associated spatial niches across the pseudotime, identifying G1 niche as the hypothetical starting point. **f** CytoTRACE score estimation showing the scale of cytoTRACE of G1–G4 niches.

To further investigate the spatial heterogeneity of epithelial niches including the normal gland and tumor compartments, we analyzed the subpopulation of epithelial loci. The epithelial niches were further divided into 4 normal gland (G1-G4) and 5 tumor (T1-T5) subpopulations (Fig. 2a, b). We observed that the different subpopulations showed distinct distribution in tissues. For example, G1 niches were predominantly located at the bottom of intestinal glands, while G3 niches were situated at the top. As the glandular epithelial cells in the bottom of colonic crypts primarily mediate proliferation, whereas those in the top surface epithelium are differentiated [12], we identified G1 as proliferated epithelial niches and the G3 as differentiated epithelial niches. Moreover, G2 niches were mainly located in the distal colon, while G4 niches specifically existed in tumor samples, adjacent to tumor, which we identified as distal epithelial niches and peritumoral epithelial niches, respectively. In addition, the tumor populations (T1–T5) also exhibited unique spatial distributions across the tissue sections. Specifically, T3 and T5 were located at the leading edge of tumor, and T1 was predominantly located in the central tumor region and adjacent to the para-carcinoma G4 niches, while T2 and T4 were interspersed within the interior of the tumor (Fig. 2b). These results indicate that the malignant epithelial niches comprise of multiple subtypes of epithelial cells with different spatial distribution and unique transcriptional features, which support maintaining tissue homeostasis and influencing the initiation and progression of tumors.

The spatial context of gene expression is critically important for tissue functionality, tissue architecture formation, and pathological changes. To further explore the functionality of distinct niches, we initially conducted Gene Ontology (GO) Enrichment analysis on normal epithelial niches (Fig. 2c). Analysis showed that G1 niche exhibited unique transcriptional programs in comparison to the remaining niches. The functional enrichment of G1 niches primarily involved in the pathways associated with cellular migration and extracellular matrix adhesion. The transcriptional features of the remaining three niches were relatively similar, predominantly mediating the pathways related to protein synthesis and processing, such as ribosomal protein synthesis assembly. To further elucidate the interplays among these niches, we conducted pseudotime trajectory analysis, uncovering a crucial involvement of G4 niches in the differentiation program, particularly from the G1 to G3 (Fig. 2d and e). CytoTRACE analysis further validated this observation, showing a gradual decrease in differentiation potential from G1 to G3 (Fig. 2f). This finding was consistent with the fact that the crypts at the bottom of epithelium consist of intestinal stem cells that gradually differentiate into mature epithelial cells at the top, forming a complete glandular structure [13]. Our aforementioned description similarly identified G1 niches as being associated with epithelial proliferation and G3 niches with epithelial differentiation. Notably, we observed an elevated differentiation potential from G4 to G3 (Fig. 2f). Given that the G4 niches represented a pre-state of G3 during the tissue differentiation progress and was located in adjacent non-tumor tissue, we speculated that tumorigenesis may originate from G4, representing an abnormal differentiation progress with a failure to reach the normal endpoint of the differentiation spectrum. This could mark the onset of an impaired differentiation program during epithelium formation at the top of the intestinal glands.

### Identification of the intratumor spatial heterogeneity

**Fig. 3:**
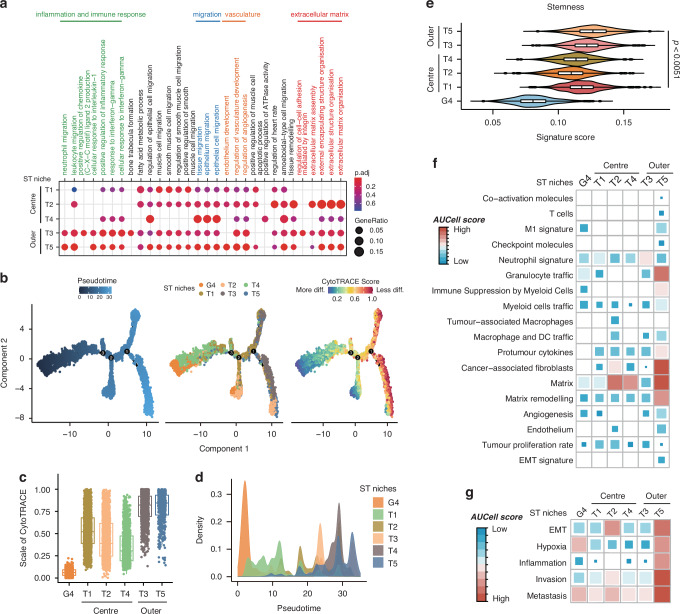
Intratumor heterogeneity in CRCs. **a** Functional enrichment analysis (GO) defining spatial niches T1–T5, presented as a dot plot. **b** Pseudotime trajectory analysis showing tumor evolution ordered in a branched pseudotime trajectory. Spatial niches are colored by pseudotime (left panel), niche type (middle panel), and CytoTRACE score (right panel). **c** CytoTRACE score estimation showing the scale of cytoTRACE of niches G4, T1–T5. **d** Distributions of tumor-associated spatial niches across the pseudotime, identifying niche G4 as the hypothetical starting point. **e** Stemness score estimation depicting the stemness of niches G4, T1–T5. Data are presented as mean ± SEM. *p* < 0.0051 by two-sided Wilcoxon rank-sum test. **f** AUCell score estimation indicating the tumor-related signaling activities within ST niches G4, T1–T5. **g** AUCell score estimation revealing a pronounced activity pattern of metastasis-related signaling within the distinct niches G4 and T1 to T5.

To gain a deeper insight into the involvement of different subpopulations of tumor cells in tumor progression and pathology, we analyzed the characteristics of populations of T1 to T5 through GO Enrichment Analysis (Fig. 3a). Notably, inflammation and immune-related pathways and extracellular matrix-related signaling pathways were enriched in T3 and T5 niches, revealing the spatial transcriptome diversity within CRC tissues. To further elucidate the tumor progression, we employed pseudotime trajectory analysis and monitored the CytoTRACE score over pseudotime (Fig. 3b and c). We applied the G4 niches as the starting point for tumor evolution and observed the peak signal of T1 to T5 was sequentially distributed across pseudotime (Fig. 3d), which was consistent with their spatial positioning. Moreover, we found that the CytoTRACE score and stemness score increased progressively from central niches (T1/T2/T4) to outer niches (T3/5), indicating a rising de-differentiation potential and enhanced cancer stemness throughout tumor progression, following a “center-periphery” mode (Fig. 3c and e). We further validated our findings through AUCell analysis, revealing that the tumor outer layer niches (T5) exhibited a strong association with matrix remodeling, granulocyte traffic, cancer-associated fibroblasts, a moderate association with neutrophils signature, and pronounced features of invasion and metastasis (Fig. 3f, g). These results suggest that the tumor niches at leading edge may mainly play a role in the TME remodeling (CAFs and neutrophils), thereby facilitating tumor invasion. Furthermore, these observations prompt two key questions: which type of cancer cell is responsible for the high stemness of CRC in niches at leading edge, and how do these tumor cells with elevated stemness contribute to TME remodeling?

### CD44^+^ tumor cells located at the leading edge of tumor

**Fig. 4:**
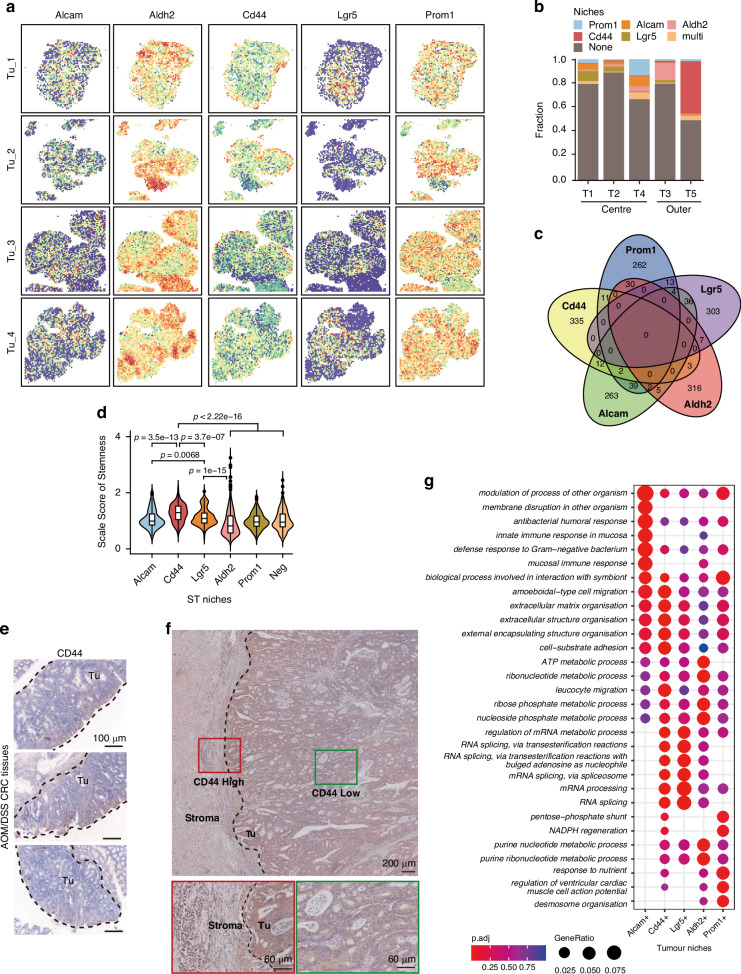
Spatially heterogeneity of stemness-related gene expression in CRC. **a** Distribution of the putative CSC gene-expressing niches of five CSC markers in four tumor sections. **b** The proportion of CSC gene-expressing niches in the ST niches of T1-T5. **c** Venn diagrams showing the numbers of five putative CSC gene-expressing niches (identified as top 10% spots with indicated CSC marker expression) in four tumor sections. **d** Stemness score estimation depicting the stemness of five putative CSC gene-expressing niches. Data are presented as mean ± SEM. *P* values were calculated using a two-sided wilcoxon rank-sum test. **e** Immunohistochemistry (IHC) staining displaying the spatial distribution of CD44 across the tumor region in CRC tissue sections from AOM/DSS mouse. Scale bar, 100 μm. **f** IHC staining displaying the spatial distribution of CD44 across the tumor region in CRC tissue sections from patients. Scale bar, 200 μm /60 μm in sect. **g** Functional enrichment analysis (GO) defining five putative CSC gene-expressing niches, presented as a dot plot.

Cancer stem cell (CSC)-like properties, characterized by both self-renewal and differentiation capacity, referred to as “high stemness”, exhibit significant diversity in their phenotype and functional roles during tumor development and progression [14]. To characterize the tumor cells responsible for high stemness in niches at leading edge, five classical CSC-related genes (Alcam, Aldh2, CD44, Lgr5, and Prom1) were selected to assess their spatial distribution patterns within the tumor (Fig. 4a–c) [15–19]. We ranked the expression levels of each CSC marker gene across all ST spots in each tissue section and defined the top 10% as CSC gene-expressing niches. We revealed distinct spatial patterns: Lgr5-positive niches were predominantly localized in the central areas of the tumor, Alcam- and Prom1-positive niches were dispersed throughout the tumor regions, while CD44-positive niches were predominantly distributed in the outer regions (Fig. 4a and b). Interestingly, we noted minimal overlap in the spatial distribution of these marker genes (Fig. 4c). Tumors originate from multiple colonies, with each colony displaying a unique CSC gene expression profile that contributes to the observed heterogeneity in CSCs. This spatial heterogeneity is a key manifestation of the phenomenon, as different CSCs exhibit distinct spatial distributions within tissue sections. These distributions reflect the polyclonal nature of tumors and suggest that tumor subclones in specific spatial niches may mediate diverse biological functions. Furthermore, we analyzed the stemness scores of these CSC gene-expressing niches and found that CD44-positive niches exhibited the highest stemness across all ST niches (Fig. 4d), suggesting that CD44-positive niches may represent the CSC niches in our colorectal cancer tissues. Additionally, we conformed the outer-expressing pattern of CD44 by immunohistochemistry (IHC) staining in both mouse and human CRC tissue sections (Fig. 4e and f), revealing a distinct spatial expression of CD44 at the tumor edge region. We further compared the functional differences among these niches characterized by positive genes related to stemness. CD44-positive niches exhibited stronger ECM organization and cell-substrate adhesion activities, which is consistent with the observation that the peripheral niches T5 showed elevated matrix remodeling and EMT activity (Fig. 3f and g). In contrast, Lgr5-positive niches displayed enhanced RNA splicing activity, while Aldh2- and Prom1-positive niches were primarily characgterized by higher metabolic activity (Fig. 4g). Notably, Alcam-positive niches also demonstrated a focus on ECM organization and adhesion pathways, but unlike CD44-positive niches, they showed significant activation of intestinal mucosal immunity-related signaling pathways and did not engage in purine metabolism pathways. This highlights the heterogeneity between Alcam-positive and CD44-positive niches. Given the profound impact of purine metabolism on tumor proliferation and metastasis, we propose that, in addition to ECM remodeling, cells at the tumor’s leading edge might adopt multiple pathways to enhance their metastatic potential [20, 21]. Overall, these results indicated high stemness of tumor peripheral is due to a predominant localization of CD44^+^ CSCs in this area.

### Characterization of the spatial distribution of cells in the tumor microenvironment

**Fig. 5:**
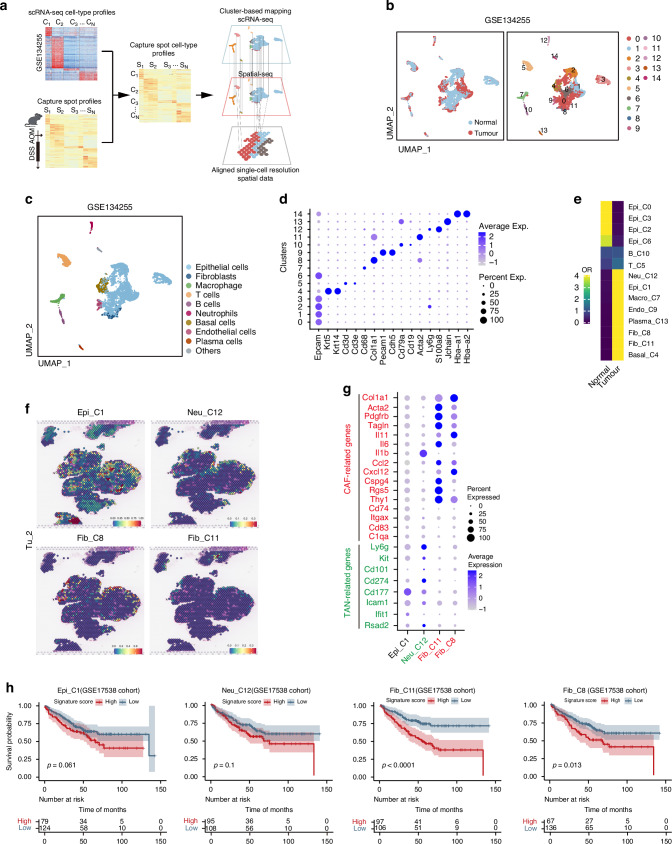
Integrative analysis of spatial and single-cell transcriptomics data identified 9 distinct cell clusters in mouse colonic epithelium and CRC. **a** Schematic representation of the cluster-based mapping involving the integration of scRNA-seq data and ST data to align single-cell resolution ST data. **b** UMAP plot showing scRNA-seq analysis of normal colonic epithelium and AOM/DSS-induced CRC tissues from GEO dataset (GSE134255), colored by sample type (left panel) and 15 cell clusters (right panel). **c** Identification of nine cells populations in normal epithelium and CRC primary tumor from the AOM/DSS mouse model, based on scRNA-seq data from GEO dataset (GSE134255), visualized by a UMAP plot. **d** Biomarkers used for cell annotation of scRNA-seq. **e** Heatmap showing the ORs of eight cells populations (integrated from 14 clusters, C0–C13) occurring in normal colon and CRC tissues, respectively. **f** Four cell populations specifically distributed in the tumor edge (Epi_C1, Neu_C12, Fib_C8, and Fib _C11) for the Tu_2 section. **g** Markers of Cancer-associated fibroblasts (CAFs) and Tumor-associated neutrophils (TANs) used for identification of cell populations (Neu_C12, Fib_C8, and Fib _C11, respectively), presented as a dot plot. **h** Kaplan-Meier survival analysis for colon cancer patients in GEO cohort (GSE17538), grouped into high (top 50%) and low (bottom 50%) Epi_C1/Neu_C12/Fib_C11/Fib_C8 signature scores, respectively. *P* values were measured by Log-rank test.

CD44-positive CSCs located at tumor leading edge exhibit an EMT phenotype and possess the capability for distant metastasis (Fig. 3g). Since the TME plays a crucial role in tumor migration [22], we aimed to clarify the components of TME close to the leading edge of tumor. Our strategy for specifying the cell types was to integrate the scRNA sequencing and ST sequencing data to overcome the relative low resolution of ST sequencing (Fig. 5a). We first analyzed previously published scRNA sequencing data from wildtype mouse colon epithelium and AOM/DSS-induced colorectal cancer (GSE134255) [23]. A total of 7196 cells were included in the analysis, with 3290 cells from the Normal group and 3,906 cells from the Tumor group. This analysis generated a unified UMAP plot containing 15 distinct cell clusters (Fig. 5b). We annotated each cluster based on transcriptional signatures: Epithelial cells (Epi), Fibroblasts (Fib), Macrophages (Macro), T cells (T), B cells (B), Neutrophils (Neu), Basal cells, Endothelial cells (Endo) and Plasma cells (Fig. 5c and d). Notably, Cluster 14 was identified as potentially representing red blood cell contamination based on the biomarker gene expression and was thus excluded from further analyses (Fig. 5d). Odds ratio (OR) analysis revealed the following significantly enriced tumor-associated cell populations: Epi_C1 (referring to the Cluster 1 within the epithelial subpopulation), Neu_C12, Macro_C7, Plasma_C13, Endo_C9, Fib_C8, Fib_C11 and Basal_C4 (Fig. 5e). Then, using scRNA-seq as a reference, we predicted these spatial cell-type distribution in our ST slides (Fig. 5a). Accordingly, Macro_C7, Plasma_C13 and Endo_C9 clusters exhibited a diffused distribution within the tumor region (Supplementary Fig. 2a), while Epi_C1, Fib_C8, Fib_C11, and Neu_C12 clusters showed similar patterns, predominantly localizing in the tumor’s edge region (Fig. 5f; Supplementary Fig. S2b).

Since the fibroblasts and neutrophils are key cellular components in TME, we further classified the type of CAFs and TANs located at the tumor-leading edge. By analyzing the expression of marker genes associated with distinct fibroblast and neutrophil subtypes, we identified high levels of Col1A1 and IL-11 in Fib_C8, elevated expression of PDGFRβ and Acta2 in Fib_C11, and high expression of Ly6G, CD101, and PD-L1 in Neu_C12. We then classified these populations as iCAF-like cells, myCAF-like cells, and pro-tumorigenic N2 TANs, respectively (Fig. 5g). Subsequently, we identified distinct gene signatures for these cell populations close to the leading edge. To clarify the clinical relevance of the distribution patterns, the cell populations were tested in a CRC cohort and showed some correlations with a poorer prognosis. In particular, the level of two CAF populations was significantly associated with an unfavorable outcome (Fig. 5h). Collectively, we show a characteristic spatial feature in CRC TME close to the leading edge of tumor, which facilitates tumor growth.

### Analyses of communications between cells around the tumor leading edge

**Fig. 6:**
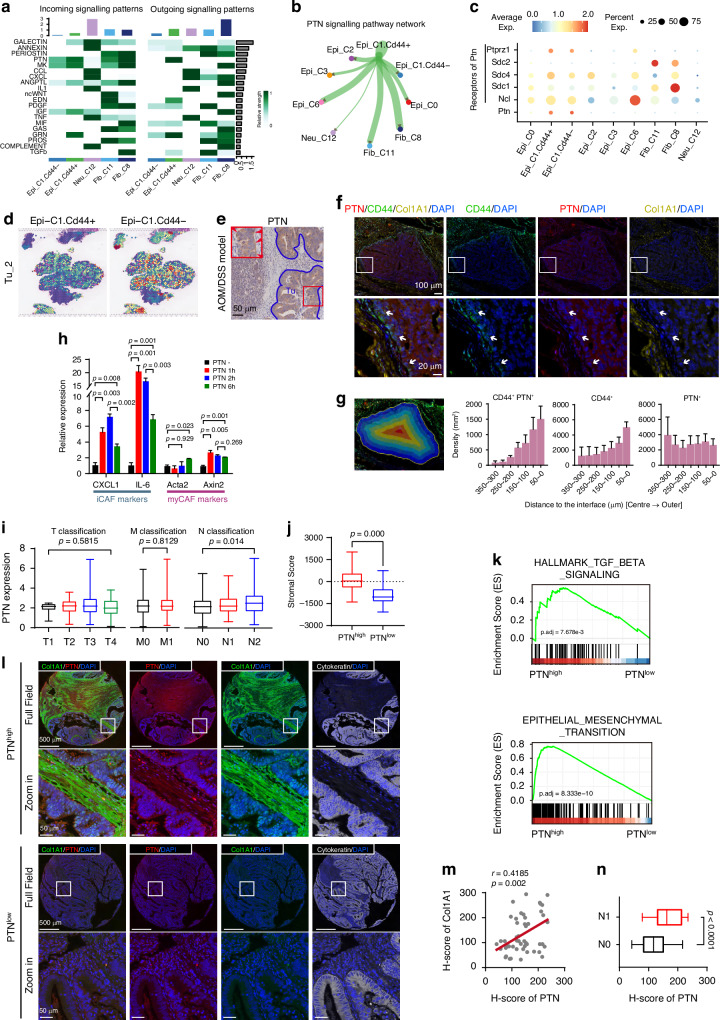
Single-cell spatial-seq data revealed tumor cell-derived PTN in CAFs phenotype, microenvironment remodeling, and CRC metastasis. **a** Contribution of the top 20 pathways identified to the incoming (left panel) and outgoing (right panel) signals among the cell populations specifically distributed in the tumor edge region, analyzed using CellChat. **b** Intensity of cell-cell interactions in the PTN signaling pathway, visualized as a circle plot. **c** The expression patterns of PTN signaling-related ligands and receptors in different cell populations, visualized as a dot plot. **d** The Epi_C1_CD44^+^ cells were specifically distributed in the tumor edge compared to Epi_C1_CD44^-^ cells in the Tu_2 section. **e** IHC staining displaying the spatial distribution of PTN across the tumor region in CRC tissue sections from AOM/DSS mouse. The red rectangle indicated the zoomed-in view. The red arrow signified PTN highly expressed in the tumor edge region. Scale bar, 100 μm. **f** Multi-color immunofluorescence (IF) staining displaying the spatial distribution of CD44 (green), PTN (red), and Collagen I (Col1A1) (light brown) across the tumor region in CRC tissue sections from patients. The white rectangle indicated zoomed-in view. White arrows signified PTN highly expressed tumor cells in the tumor edge region. 4’,6-diamidino-2-phenylindole (DAPI) (blue) staining indicated nuclei. Scale bar, 100 μm /20 μm in sect. **g** Left, the representative illustration of the strategy for tumor tissue region segmentation analysis. The tumor regions are consistent with those shown in Fig. 6f; right, the bar graph displaying the distribution density of CD44^+^PTN^+^, CD44^+^, and PTN^+^ tumor cells from the tumor center to the tumor edge. The x-axis indicates the distance from the tumor edge, with 0 μm representing the tumor boundary. This analysis includes data from six tumor regions. Data are presented as mean ± SEM. **h** qRT-PCR analysis showing iCAF and myCAF marker expression induced by recombinant PTN protein. CAFs were isolated from fresh CRC tissues and treated with PTN (50 μg/ml) for indicated time period. Data are presented as mean ± SEM (*p* value from unpaired Student’s *t*-test). **i** Correlation of PTN expression with clinicopathological staging characteristics in Colon adenocarcinoma (COAD) patients from TCGA database. Left panel, T classification; middle panel, M classification; right panel, N classification. Data are presented as mean ± SEM (*p* value from unpaired Student’s *t*-test). **j** Distribution of StromalScore with differentiated PTN expression in COAD patients from TCGA database. Data are presented as mean ± SEM (*p* value from unpaired Student’s *t*-test). **k** Gene Set Enrichment Analysis (GSEA) analysis (Hallmark gene sets) of COAD patients from TCGA database, showing the pathways associated with the high-expressed PTN group (*p* value from log rank test). **l** Multi-color IF staining of PTN (red), Col1A1 (green), and cytokeratin (white) in CRC tissue sections from 51 COAD patients. The white rectangle indicated zoomed-in view. 4’,6-diamidino-2-phenylindole (DAPI) (blue) staining indicated nuclei. Scale bar, 500 μm/50 μm in sect. See Table S2 for Characteristics and clinical data of patients. **m** Correlation between PTN expression in tumor cells and Col1A1 expression in stroma across 51 CRC tissue sections from COAD patients, visualized as a scatter plot. Each dot represents a separate tissue section. *r* = 0.4185, *p* = 0.002 by Linear regression and Spearman test. **n** Differentiated expression of PTN between COAD tissues with or without metastatic lymph nodes based on pathological diagnosis (N1 = 25, N0 = 26, respectively). Data are presented as mean ± SEM (*p* value from unpaired Student’s *t*-test).

Cells in close proximity are presumed to engage in intercellular communication to fulfill cellular function in an interactive niche. As presented, there were the spatial enrichment of cell populations, including tumor cells exhibiting high CD44 expression, CAFs and TANs at the tumor’s edge (Fig. 5f). To further investigate how CD44^+^ tumor cells remodel the TME, we analyzed the communications between these cells. We evaluated the incoming and outgoing signaling patterns by CellChat analysis in these cell clusters (Fig. 6a and Supplementary Fig S3a) [24]. For the Epi_C1 clusters, we further classified cells into CD44-positive and CD44-negative clusters based on CD44 expression, referring to them as CD44^+^ and CD44^-^ Epi_C1, respectively, to explore the specific functions of CD44^+^ tumor cells at the leading edge of CRC tissues. A strong pleiotrophin (PTN) signaling from CD44^+^ Epi_C1 clusters was received by CAF clusters (Fib_C8 and Fib_C11) (Fig. 6b, c). It is worth note that CD44^-^ Epi_C1 clusters also exhibited a moderate level of PTN, although there was no definite clue show that CD44^-^ Epi_C1 clusters had a PTN signaling crosstalk with CAF clusters based on CellChat analysis (Fig. 6c). Since CD44^-^ Epi_C1 clusters were located closer to the interior of the tumor, away from infiltrated immune cells and stromal cells, compared to CD44^+^ Epi_C1 clusters (Fig. 6d and Supplementary Fig S3b), we speculated that PTN from CD44^-^ Epi_C1 may interact with other cell clusters rather than TANs or CAFs at the tumor leading edge.

Next, we examined the spatial distribution of PTN. IHC staining results confirmed that the level of tumor cell-expressed PTN was much higher in leading edge than that in central area of tumor regions (Fig. 6e). The fluorescence staining results for CD44, PTN and Collagen revealed that CD44^high^-PTN^high^ tumor cells was observed not only specifically at the leading edge, but also near fibroblasts infiltrated within tumor microenvironment (Fig. 6f). Further, we found that the density of CD44^+^ and CD44^+^PTN^+^ tumor cells increased progressively from the tumor center towards the periphery, supporting our previous findings that CD44^+^ cells at the tumor leading edge secrete high levels of PTN (Fig. 6g). In contrast, PTN^+^ cells did not exhibited significant spatial patterns, suggesting that, in addition to CD44^+^ tumor cells, other cells may contribute to PTN signaling activation and regulation in a manner independent of tumor edge CAFs and neutrophils (Fig. 6b and g).

PTN is a PDGF-inducible cytokine, secreted by various human cancer cells and promotes tumorigenesis, tumor growth, as well as tumor angiogenesis [25–27]. Highly expressed PTN strikingly increased the synthesis of collagen and elastin in breast cancer derived from MMTV-PyMT transgenic mice [28], but the detailed mechanism was unclear. Thus, we speculate that PTN could facilitate the remodeling of the stroma via activating CAFs in CRC. To verify our hypothesis, we isolated colonic fibroblasts from fresh tumor tissues and treated them with recombinant PTN at indicated time period (Fig. 6h). The effect of PTN on induction of iCAF- and myCAF-related phenotype was examined by qRT-PCR. The iCAF markers, CXCL1 and IL-6, were strikingly increased after PTN stimulation for 1 hour and moderately decreased after 6 hours; in contrast, canonical myCAF markers Acta2 and Axin2 showed comparable expression at 1 hour but increased by 6 hours, suggesting PTN predominantly induces a transition to iCAF-related phenotype, with moderate myCAF-related phenotype induction.

Together, our results here showed the CD44^+^ CSCs-mediated PTN signaling may part be responsible for remodeling TME, driving the differentiation of CAFs into iCAF-like and myCAF-like cells, with a predominant effect on the former. This transition may subsequently facilitate the recruitment of pro-tumorigenic TAN close to the leading edge of tumor.

### Tumor-derived PTN that is responsible for phenotypic plasticity of CAFs and tumor progression

Tumor microenvironment remodeling is thought to be a prerequisite for tumor invasion and metastasis [29]. We wondered whether the CD44^+^ tumor cells with high stemness gained the enhanced invasive and metastasis potential through a PTN-dependent and CAFs-associated manner. We first determined the relationship between the PTN expression and clinicopathological characteristics with the samples of colon adenocarcinoma (COAD) bulk transcriptome dataset from The Cancer Genome Atlas (TCGA) cohort. The level of PTN showed a positive correlation with N classification of TMN stages, but no correlation with T or M classification (Fig. 6i), which was consistent with the previous report that CAFs promote lymph node metastases [30]. We then compared the StromalScore between PTN^high^ and PTN^low^ COAD samples, and found a dramatic decline of StromalScore in the PTN^low^ samples (Fig. 6j). Given that stroma activity is one of the crucial factors affecting tumor invasion and metastasis, we performed gene set enrichment analysis (GSEA) to investigate the differential enrichment of metastasis-associated pathways between PTN^high^ and PTN^low^ groups. As expected, the TGFβ signaling and epithelial mesenchymal transition program were enriched in the PTN^high^ group (Fig. 6k), suggesting that PTN expression may increase the metastasis potential [27, 28].

Finally, to investigate the clinical relevance of our findings, we evaluate the correlations among PTN expression, stroma diversity and metastasis status in colorectal cancer samples. The multi-color immunofluorescence staining revealed that tumor cells exhibiting higher PTN expression were closely associated with fibroblasts infiltration in stromal regions, showing a positive correlation with tumor metastasis (Fig. 6l–n; Supplementary Table S2). Taken together, these data suggest that PTN may partly contribute to stromal remodeling and play a significant role in tumor metastasis. Targeting PTN might be a promising strategy for the treatment of metastatic colorectal cancer.

## Discussion

In this study, we systemically investigated the tumor heterogeneity and microenvironment remodeling in the AOM/DSS-induced CRC mouse model by integrating scRNA-seq and ST-seq. Our findings revealed a distinctive layered ring-like organization within CRC tissues, illustrating transcriptional and cellular spatial variability corresponding to tissue depth. Specifically, we identified CD44^+^ tumor cells, as well as CAFs and TANs infiltrated in TME, which exhibited a specific distribution in tumor edge region. Mechanistically, CD44^+^ tumor cells at the edge can secrete PTN, a PDGF-inducible cytokine, which acts as a chemokine, aggregates CAFs, and leads to their transformation to iCAF-like or myCAF-like cells. This process may contribute to the establishment of a pro-survival TME, ultimately facilitating tumor progression.

Tumor heterogeneity refers to variations in genetic background or biological behavior within a tumor as it proliferates through multiple rounds of cell divisions. These variations can manifest as diverse phenotypes, such as, tumor growth patterns, levels of differentiation, invasion capacities, metastatic potential, and responses to treatments. As tumor heterogeneity is often associated with clinical outcome, it is essential to identify the differentiated expression profiles and molecular events within the tumor for overcoming treatment resistance and advancing precision medicine. We here thoroughly explored the spatial heterogeneity landscape of CRC using ST-seq, identifying a distinctive spatial heterogeneity pattern characterized by a layered ring-like structures within tumor tissues, where tumor growth follows a hierarchical diffusion pattern that progresses “from the inside out”, alongside with decreased differentiation potential and increased de-differentiation potential. Indeed, our findings not only elucidate differential gene expression across spatial regions of CRC, may also provide some new additions into the theory of tumor progression.

In this study, we utilized the AOM/DSS-induced CRC animal model for spatial research, followed by validation using public scRNA-seq data. We choose AOM/DSS-induced colorectal cancer model because it is the classical colorectal model that can simulate the progression of tumorigenesis. Despite the inherent heterogeneity in CRC data across mouse tissue sources, the selected data were consistent in terms of animal model and mouse age, and our findings were further supported by human CRC samples. While the sampling included normal (N) and tumor (Tu) groups from different regions of the colon (with the N group comprising the entire colon segment and the Tu group comprising tumor tissues predominantly localized to the distal colon, respectively)—acknowledging that DSS effects are most pronounced distally [31]—our study focused on tumor-intrinsic heterogeneity and TME remodeling, minimizing the influence of regional pathological differences. Although the AOM/DSS model does not fully replicate clinical CRC due to its random gene mutation patterns [31], we addressed this limitation by performing immunohistochemical validations using patient-derived CRC samples and confirmed our findings from the animal model through in vitro co-culture experiments with isolated CAFs. Consequently, we believe the conclusions of our study are robust and credible.

CSCs play a crucial role in driving tumor proliferation and maintaining long-term tumor vitality [32]. Interestingly, although tumors with higher stemness often exhibit increased tumor mutational burden (TMB), tumor antigenicity, and intratumoral heterogeneity, which is expected to enhance immune infiltration, there exists a prevalent negative correlation between CSCs and anti-tumor immunity [7, 33, 34]. Our results revealed that the immunosuppressive cells derived from neutrophils and fibroblasts were enriched around the tumor edge region, where the high stemness features (with CD44 as a CSC marker), further supporting the notion that the CSCs are associated with immune suppressive effect of anti-tumor response. CD44, a transmembrane adhesion glycoprotein expressed on the surface of various cancer cells and stromal cells, is not only involved in EMT, promoting tumor progression and metastasis [17], but also plays a role in the adhesion of peripheral neutrophils, which leads to neutrophil migration and recruitment [35]. This is consistent with our findings, in that the high enrichment of TANs in the fibrous capsule is associated with the TME architecture. Thus, we provide direct evidence showing that cancer stemness signals drives spatial heterogeneity of TME.

CAFs, integral constituents of the TME, play central roles in recruiting various immune cells, regulating the tumor immune response, and providing a protective shield [36]. At least two primary CAF subtypes, namely myCAF and iCAF, have been identified in pancreatic adenocarcinoma using the “-omics” technology [37, 38]. In alignment with these findings, our analysis of CRC data revealed a CAF population with high expression of Col1A1 and IL-11, and another characterized by high expression of PDGFRβ and Acta2 (Fig. 5g). Therefore, we defined these populations as iCAF-like and myCAF-like cells, respectively. Recent studies employing scRNA-seq and ST-seq have elucidated their roles in promoting tumorigenesis, with a notable example where the FAP^+^ CAFs (the activated CAFs expressing fibroblast activation protein, which promote the tumor progression) in CRC cooperated with SPP1^+^ macrophages to remodel the extracellular matrix, thereby impeding T cell infiltration [39]. Additionally, it has been reported that CAFs derived from T1 stage CRC promote the upregulation of CD44 in epithelial cells [40], suggesting a reprogramming effect of CAFs on tumors. In contrast, our study demonstrates that PTN derived from CD44^+^ tumor cells facilitates the phenotype transition of CAFs. Our findings, to some extent, support previous findings, highlighting a complex regulatory interaction between CAFs and CD44^+^ CRC cells, which may ultimately drives tumor progression. Another research published by Roelands et al. also demonstrated that myeloid cell infiltration is important during CRC formation and progression [41], aligning with our research findings. Although our current validation is preliminary, we intend to conduct additional experiments in the future to better validate the interactions between CD44^+^ CSC cells and CAFs.

In addition to investigating the biological behaviors and mechanisms of CAFs in tumors, various efforts have been directed toward therapeutic targeting of CAFs. The attempts to block FAP^+^ have shown some promises in inducing tumor necrosis and immune-responsive cytotoxicity when combined with immune checkpoint blockade in pancreatic cancer mouse models [42, 43]. Several clinical trials have also focused on CAF-targeted therapies, revealing increased immune cell infiltration and reduced tumor growth. However, the extent of survival benefits from these therapies remains to be assessed, requiring further investigation [44]. We identified tumor-derived PTN as a promoter to drive lymph node metastases in a CAF-dependent manner, potentially offering a promising target for overcoming CRC relapse. Additionally, we identified a population of tumor-associated neutrophils (TANs) at the CRC leading edge that exhibits high expression of PD-L1 (Fig. 5f). While the AOM/DSS model is neutrophil-driven and even neutrophil-dependent for tumorigenesis [45], a substantial infiltration of TANs is not surprising. However, the notable upregulation of PD-L1 within this TAN population implies a role in immunosurveillance regulation. We hypothesize that these PD-L1^high^ TANs are likely recruited and regulated by CAFs, collectively contributing to the formation of an immunosuppressive TME, thereby promoting tumor progression. Nevertheless, additional research and investigation are required for a comprehensive understanding of its therapeutic potential.

In summary, our study presents a spatial and cellular atlas of CRC using a chemically induced spontaneous colorectal cancer model, and identifies the PTN-mediated tumor progression and the diversity of CAFs within the TME. Our findings not only provide potential molecular targets for CAF-directed therapies, but also present unique insights into cancer biology.

### Limitations of the study

While we utilized the spatial transcriptomics technology for whole transcriptome sequencing of mouse CRC tumor tissues developed by 10× Genomics, the sample size limitation prevented us from capturing the entire pathological landscape of the intestinal tissue, including both the tumor and surrounding regions. As a result, we missed valuable information on disease progression at different stages, such as low-grade and high-grade dysplasia. Further exploration using additional technologies (e.g., Digital Spatial Profiling [41]) to investigate the changes in tissue morphology and gene regulation during CRC development and progression is needed.

## Supplementary information

Supplementary file

## Data Availability

Data generated in this study are available within the article and its supplementary data files and are available upon request from the corresponding author. The datasets generated in this study are publicly available in BioProject at PRJNA1093360. The data generated by others and analyzed in this study were obtained from GEO at GSE134255 and GSE17538.
