# Supplementary material for: CD44+ cells enhance pro-tumor stroma in the spatial landscape of colorectal cancer leading edge
Source: Br J Cancer. 2025 Mar 12;132(8):703–15. doi: 10.1038/s41416-025-02968-9 (PMC11997037; doi:10.1038/s41416-025-02968-9)
Supplement: Supplementary file 1 — Supplementary file [file 41416_2025_2968_MOESM1_ESM.pdf]

SUPPLEMENTARY FIGURES AND TABLES

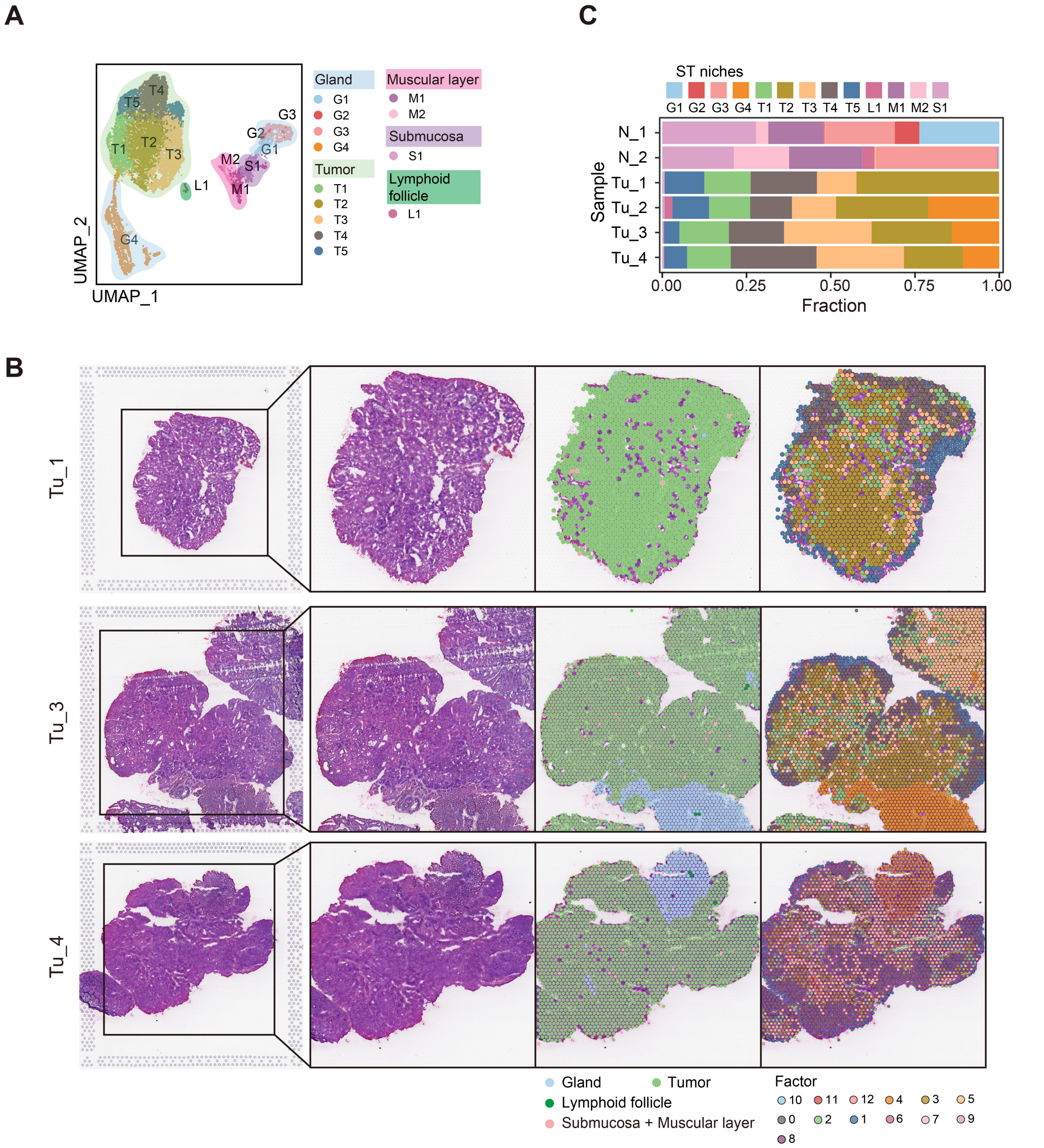

Supp. Fig. S1. Exploration of CRC architecture with ST. Related to Figure 1.

A UMAP (Uniform Manifold Approximation and Projection) plot illustrating 13 color-coded clusters from all sections, categorized as G (gland)/T (tumor)/L (lymphoid follicle)/M (muscular layer)/S (submucosa).

B Left panel, H&E staining; right panel, distribution of spatial clusters for each section, except N_1, N_2 and Tu_2 as shown in Figure 1.

C The fraction of spatial clusters in each section, categorized as G (gland)/T (tumor)/L (lymphoid follicle)/M (muscular layer)/S (submucosa).

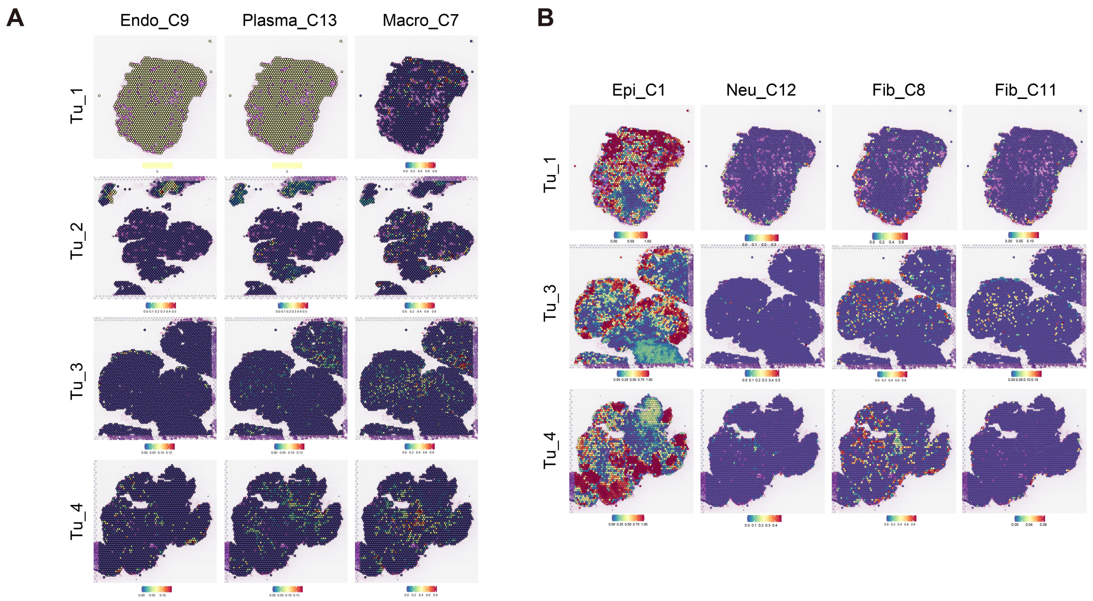

Supp. Fig. S2. Characterization of cells at the tumor edge. Related to Figure 5.

A The spatial distribution of cell populations (Endo_C9, Plasma_C13 and Macro_C7) for each section.

B Four cell populations specifically distributed in the tumor edge (Epi_C1, Neu_C12, Fib_C8, and Fib _C11) for each section, except Tu_2 as shown in Figure 4.

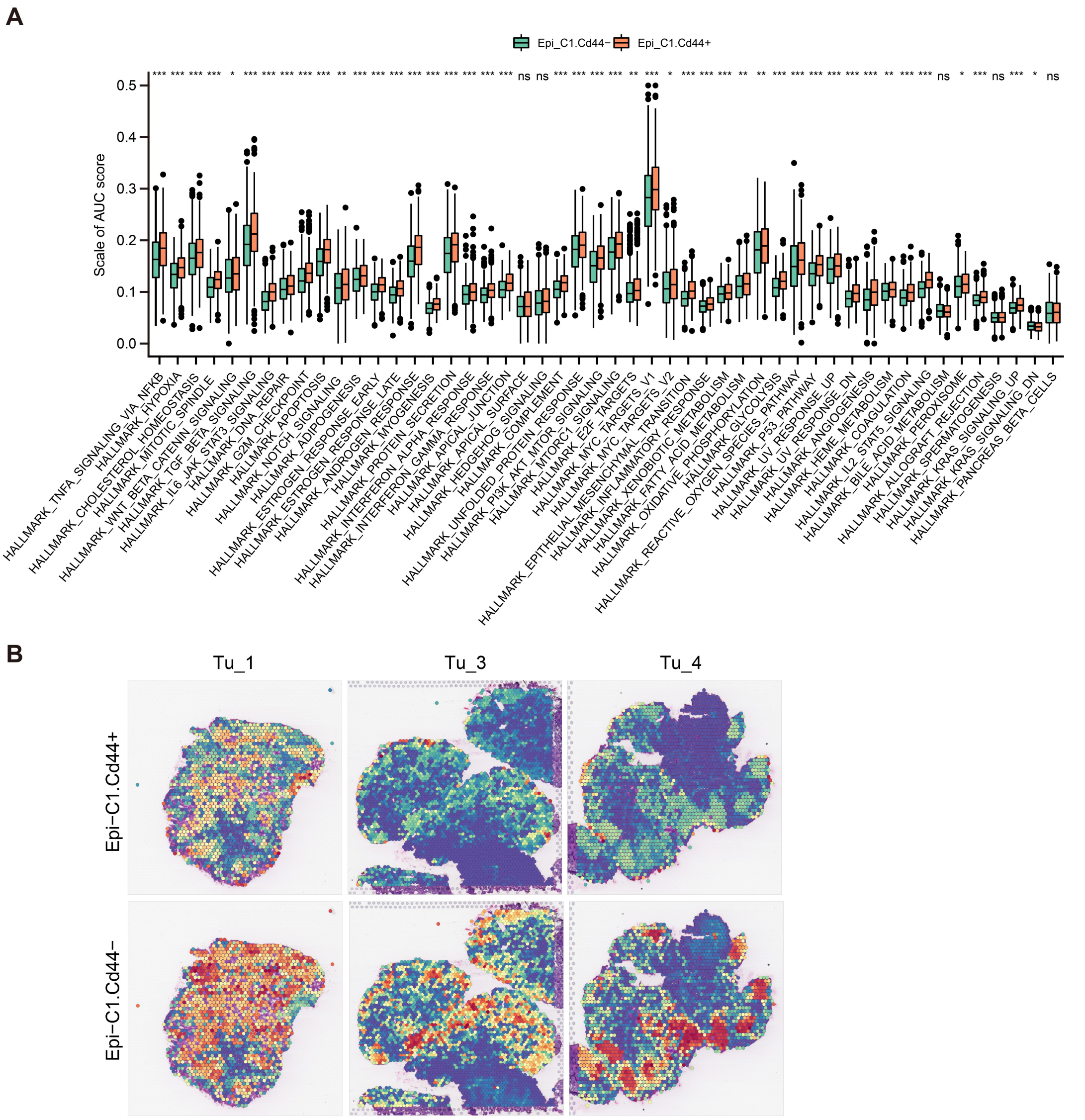

Supp. Fig. S3. Identification of CAF-TAN communications at the tumor edge. Related to Figure 6.

A Differential activation of Hallmark pathway in the CD44-positive Epi_C1 cluster compared to the CD44-negative Epi_C1 cluster.

B The distribution of Epi_C1_CD44^{+} Epi_C1_CD44^{-} in tumor sections, except Tu_2 as shown in Figure 6.

Supp. Table S1. Summary of Spatial Transcriptomics Sequencing Data for Each Sample

Sample ID	Sample Type	Number of Spots Under Tissue	Median Genes per Spot	Number of Reads	Mean Reads per Spot	Total Genes Detected	Median UMI Counts per Spot
N_1	Normal	659	4356	243084548	252949.5817	18684	20569
N_2	Normal	687	4947	233190161	223790.9415	18856	22374
Tu_1	Tumor	1397	6325	219953329	138596.9307	19833	27032
Tu_2	Tumor	1414	5963	179755537	115747.2872	20230	27792
Tu_3	Tumor	3367	4871	167589135	49378.05981	20411	18181
Tu_4	Tumor	2187	5530	167949164	75482.77034	20196	22550

Abbreviation: UMI, Unique Molecular Identifier.

Supp. Table S2. Clinical and Pathological Data of 51 patients with CRC.

Patient ID	Gender	Age	Tumor Type	Site	Grade	Size (cm)	TMN	Stage	Microsa-tellite
1	Male	46	Ad	transverse colon	G2	4×2.5×1.0	T3N0M0	IIA	MSS
2	Male	41	Ad	sigmoid colon	G2	4×1	T3N1M1	IVB	MSS
3	Male	60	Ad	rectum	G2	3.5×2.0×1.5	T3N0M0	IIA	MSS
4	Male	65	Ad	rectum	G2-G3	5×3×1.9	pT3N0M0	IIA	MSS
5	Male	49	Ad	Colon	G2-G3	4.5×4.3×1.7 1.5×1×0.5	T3N0M0	IIA	MSI-L
6	Female	55	Ad	Colon	G2	3.5×3.5×3	T3N0M0	IIA	MSS
7	Male	64	Ad	Right hemicolon	G3	5×4×4.8	T4N0M0	IIA	MSS
8	Male	45	Ad	rectum	G1-G2	2.5×1.5×1.3	T3N2M0	IIIB	MSS
9	Male	61	Ad	Left hemicolon	G2	4×3.7×4	T3N2M1	IVB	MSS
10	Female	71	Ad	Right hemicolon	G2	8×7×5.5	T3N0M0	IIA	MSS
11	Male	55	Ad	sigmoid colon	G2	4×4×1	T3N0M0	IIA	MSS
12	Male	75	Ad	sigmoid colon	G2-G3	4.5×3.5×2.5	T3N0M0	IIA	MSS
13	Female	61	Ad	sigmoid colon	G2	6×6×2.5	T3N0M0	IIA	MSS
14	Male	33	Ad	Right hemicolon	G2-G3	6×4.6×1.5	T4N11a	IVA	MSS
15	Male	52	Ad	sigmoid colon	G1	6×5×3	T3N0M1b	IVB	MSS
16	Female	29	Ad	sigmoid colon	G1-G2	7×5×9	T4N1M0	IIIB	MSS
17	Male	63	Ad	sigmoid colon	G1-G2	7×4×2	T3N1M0	IIA	MSS
18	Male	68	Ad	rectum	G2	9×6×2.5	T3N1Mx	IIB	not detected
19	Male	60	Ad	sigmoid colon	G2	4×3.5×1.7	T2N0M0	IIA	MSS
20	Male	62	Ad	rectum	G2	4×2.5×1.7	T2N0M0	IIA	MSS
21	Male	58	Ad	descending colon	G2	10×8×6	T4N1M0	IIIB	MSS
22	Female	72	Ad	rectum	G2	6×4×1.5	rT4bNxM0	IIIC	MSS
23	Female	54	Ad	rectum	G2	4×1.5×1.3	T3N0M0	IIA	MSS

24	Female	58	Ad	rectum	G2	3×2.1×1 1.5×0.8×1.2	T3N1M0	IIA	not detected
25	Male	68	Ad	Colon	G2	4×2.5×1.5	T2N0M0	IIA	MSS
26	Female	63	Ad	sigmoid colon	G2	3.5×3.4×1.5	T3N0M0	IIA	MSS
27	Male	46	Ad	Right hemicolon	G1-G2	5.5×5×3.2	T3N1Mx	IIB	MSS
28	Male	67	Ad	sigmoid colon	G2	2.3×1.8×1.2	T3N2Mx	IVB	MSS
29	Male	42	Ad	rectum	G3	2.5×2.5×0.5	T3N2Mx	IVB	MSI-H
30	Male	59	Ad	rectum	G2	3×3×0.8	T3N0M0	IIA	MSS
31	Female	69	Ad	Left hemicolon	G2-G3	3.5×2.5×1.6	T4N1M0	IIB	MSS
32	Male	69	Ad	rectum	G2-G3	4×2.3×1.8	T3N1M0	IIIB	MSS
33	Female	55	Ad	sigmoid colon	G2	5×2.3×1.9	T3N0M1	IV	MSS
34	Male	58	Ad	ileocecum	G1-G2	4×4×4	T3N2bM1	IVB	MSS
35	Female	66	Ad	sigmoid colon	G1-G2	3×2.5×0.9	T4aN2M0	IIIC	MSS
36	Female	64	Ad	Right hemicolon	G1-G2	6×5×2	T4N2M0	IIIB	MSS
37	Male	31	Ad	descending colon	G2	6×2.5×1.5	T4bN0M0	IIC	MSS
38	Male	67	Ad	rectum	G1-G2	3.5×3×1	T3N1M0	IIA	MSS
39	Male	76	Ad	Right hemicolon	G2	6×3.5×1.5	T3N0Mx	IIA	MSS
40	Female	51	Ad	Right hemicolon	G2-G3	4×3×1	T3N2M1	IVB	MSS
41	Male	58	Ad	rectum	G2	5.5×3.5×1.5	T3N0M0	IIA	MSS
42	Female	49	Ad	Right hemicolon	G2-G3	11×7×2.5	T3N1M0	IIA	MSI-H
43	Male	64	Ad	rectum	G1-G2	5×5×1	T3N1M0	IIA	MSS
44	Female	40	Ad	sigmoid colon	G1-G2	6×5×1	T3N0M0	IIA	not detected
45	Female	63	Ad	sigmoid colon	G2-G3	3.5×3×2.7	T3N2M1	IVB	MSS
46	Female	34	Ad	Right hemicolon	G2	3×1.8×1	pT4bN0M0	IIC	not detected
47	Male	51	Ad	sigmoid colon	G2	6×4×5	T2N0M0	IB	MSS
48	Male	44	Ad	Right hemicolon	G2	5×4×2	T3N0M0	IIA	MSI-H
49	Male	70	Ad	rectum	G2	3×3×0.6	T2N1M0	IIA	MSS

50	Female	67	Ad	Right hemicolon	G3	9×8×3	T3N2M0	IIIB	MSS
51	Male	51	Ad	Right hemicolon	G2	3.8×3×1.5	cT3N1M1	IVB	not detected

Abbreviations: Ad, Adenocarcinoma; MSS, Microsatellite Stable; MSI-H: Microsatellite Instability High; MSI-L, Microsatellite Instability Low.

SUPPLEMENTARY MATERIALS

Spatial transcriptome capture

Samples were collected from AOM/DSS or wildtype mice, rapidly frozen in liquid nitrogen, and embedded in OCT. 8~10 tissue slices of 10 μm thickness were obtained for RNA extraction and quality control, with only tissues having a RIN value greater than 7.0 reserved for subsequent experiments.

Before generating Visium Spatial Gene Expression libraries, the Visium Spatial Gene Expression protocol (10× Genomics, Visium Spatial Gene-Expression Reagent Kits) was employed to determine the optimal permeabilization time. Briefly, 10 μm-thick tissue slices were fixed and stained for imaging, and permeabilized for varying durations (3, 6, 12, 18, 24, and 30 minutes). The optimal conditions of an 18-minute permeabilization for tumor tissues and a 24-minute permeabilization for normal colon tissues, yielding the maximum fluorescence signal, were established. Subsequent steps included reverse transcription, second-strand synthesis and denaturation, cDNA amplification and Visium spatial gene-expression library construction, all conducted following the manufacturer's instructions (10× Genomics). The sequencing of all spatial transcriptome libraries was performed on an Illumina NovaSeq 6000 by NuoHe Bio-Tech Co., Ltd.

Spatial transcriptome (ST) data analysis

Raw sequencing reads of spatial transcriptomics were quality controlled by fastp (v0.20) and mapped to reference genome by Space Ranger (v1.2). The gene expression matrix based on spot generated after ST data processing from ST and

Visium samples were analyzed with the Seurat package (v4.1.0) in R. Some general thresholds were used to filter the expression matrix, which mainly included removing genes expressed in only three spots, removing spots with fewer than 200 genes, fewer than 1000 read counts, and more than 25% mitochondria. NormalizaData, RunPCA, RunUMAP, FindNeighbors and FindClusters are used for normalization, reduction and clustering analysis of the expression matrix based on spots. All parameters are default in addition to the resolution one and the first 30 principal components. The characteristic genes of spatial niches were identified by FindAllMakers function, setting a cutoff of *Padj* = 0.05. SpatialDimPlot and SpatialFeaturePlot are used to visualize spatial features. Pseudotime trajectory analysis was performed using Monocle 2 packages (v2.20.0) within the R software. Single-cell differentiation status was assessed by utilizing the CytoTRACER package (v0.3.3), available at https://cytotrace.stanford.edu.

scRNA-seq data processing and integrating with ST-seq

The scRNA-seq data of AOM/DSS mouse model were obtained from the publicly available GEO dataset (GSE134255). Filters were applied to retain cells with 200~3,500 genes, 1,000~70,000 UMl counts, and <15% of mitochondrial reads in each individual sample. DoubletFinder (v2.0.3) was employed for the identification and removal of doublets. Cell clusters were clustered using FindNeighbors and FindClusters, and the data were visualized using RunUMAP. Differential gene expression analysis for distinct cell clusters was performed using the FindAllMarkers

function, with a cutoff setting at *Padj* = 0.05. The TransferData function of the Seurat (v4.1.0) was used to integrate the data of scRNA-seq and ST-seq, where scRNA-seq was defined as reference and ST-seq was defined as query. The predicted spatial features were visualized using the SpatialFeaturePlot.

Survival analysis

Cohort data of colorectal cancer (CRC) patients were collected from the GEO database (GSE17538). The signature gene-sets were extracted based on cell subtypes of single-cell data by FindAllMarkers function. The score of signature gene-sets were calculated by ssGSEA methods. Survival package of R was used to perform survival analysis. Surv_cutpoint function was used to group the dataset and select the overall survival (OS) as the event parameter. Cox proportional hazards model was used to calculate Hazard ratio (HR) and 95% CI was reported. Kaplan–Meier survival curve was modeled by survfit function, and visualized by ggsurvplot function.

Cell-cell communication analysis

The analysis of cell-cell communication among specific cell groups was conducted using CellChat (v1.1.3) package. Ligand-receptor (LR) interactions were extracted from the 'CellChatDB' database (http://www.cellchat.org/cellchatdb/). The resulting cell-cell communication network was visualized through a circular plot generated by the netVisual_aggregate function. Heatmaps were used to visualize the contribution of signaling pathways to cell-cell communications by

netAnalysis_signalingRole_heatmap function.

Cancer hallmarks and gene-set enrichment score

Cancer hallmark gene sets, GO biology process gene sets and KEGG gene sets were downloaded from The Molecular Signatures Database27 (MSigDB, available at http://software.broadin-stitute.org/gsea/msigdb/). Functional state gene sets of cancer cells were downloaded from CancerSEA database (http://biocc.hrbmu.edu.cn/CancerSEA/). Knowledge-based functional gene expression signatures were collected from previous research [1]. Scoring these gene signatures was performed by R package AUCell (v1.14.0). In briefly, AUCell uses the "Area Under the Curve" (AUC) to calculate whether a critical subset of the input gene set is enriched within the expressed genes for each cell. Heatmaps were used to visualize the scores of these signature genes by R package pheatmap (v1.0.12).

TCGA data analysis

TCGA data analysis was conducted for colon adenocarcinoma (COAD). Gene expression data and clinical information were obtained from the Cancer Genome Atlas (TCGA) Data, which includes 456 tumor samples, accessible at https://tcga-data.nci.nih.gov/tcga/. For PTN expression analysis, the 456 tumor samples were categorized based on their clinical characteristics. To examine the correlation between PTN and stromal score estimates, tumor samples were classified into high or low PTN expression groups using the median expression as a reference,

followed by analysis utilizing ESTIMATE algorithm within the R (v 4.3.1). Gene Set Enrichment Analysis (GSEA) involved the utilization of 50 cancer hallmark signatures (MSigDB, H sets) and 1421 GO gene sets (MSigDB, C5 sets).

Isolation, expansion and phenotype detection of primary cancer-associated fibroblasts (CAFs)

To isolate and expand primary CAFs, 1~2mm-diameter pieces of fresh CRC tissues were plated into a 6-well culture plate, and digested with a few drops of Digestive Enzyme solution (PRECEDO, Anhui, China) for 20 min at 37°C. After carefully removing the Digestive Enzyme solution, the tissues were allowed to stew for a few minutes to attach to the bottom of the culture plate. Subsequently, the tissues were infiltrated with DMEM medium supplemented with 10% FBS and cultured for approximately 5~7 days in a 5% CO_2 incubator at 37°C. Fibroblast migration from the tissue explants was observed under a microscope. Once a substantial outgrowth of fibroblasts was achieved, the cells were harvest by trypsinization and transferred to a new 10-cm culture plate for an additional 5~7 days culture until the cell counts were sufficient for subsequent experiments. CAFs were passaged every 6~8 days while culturing in 10-cm dishes, with a total passage range of two to six.

For phenotype detection, primary CAFs were prepared in a 12-well culture plate until reaching 70-80% confluence. The cells were then treated with PTN (50 μg/ml) for 1, 2, and 6 hours, respectively. Then cells were harvested for total RNA extraction, and the phenotype was detected using a qRT-PCR assay.

qRT-PCR assay

Total RNA was extracted using TRIzol reagent (Invitrogen, 15596026), and reverse transcription was performed using PrimeScript RT Reagent Kit (TAKARA, RR047A) according to the manufacturer's instructions. The resulting cDNAs were used as templates for amplification and detection by real-time PCR using SYBR Green PCR Master Mix (Vazyme Biotech Co, Q711-02). GAPDH were used as control for normalization.

Immunochemistry staining

The procedure for the immunochemistry staining assay was previously described [2]. In briefly, individual tumors from AOM/DSS mice or patients with CRC were dissected and fixed in 4% paraformaldehyde. Samples were then baked for 1 hour at 60°C and deparaffinized. Antigens were retrieved in EDTA antigen retrieval buffer (pH 8.0) for 30 minutes. After washing with PBS containing 0.1% Tween-20, tissues were blocked with PBS containing 5% bovine serum albumin for 30 minutes at RT, followed by incubation with following antibodies overnight at 4°C: anti-CD44 (proteintech, Cat#15675-1-AP, RRID: AB_2076198), anti-PTN (ABclonal, Cat#27117-1-AP, RRID: AB_2880763). The tissues were then treated with a 3% hydrogen peroxide/ethanol solution for 15 min at RT and incubated with secondary antibodies for another 30 min at RT, followed by signal detection with diaminobenzidine solution.

All images were captured and analyzed using an InForm Cell Analysis 2.2 (PerkinElmer).

Multi-color Immunofluorescence staining

Paraffin sections from patients with CRC were baked for 1 hour at 60℃ and deparaffinized. Antigens were retrieved in EDTA antigen retrieval buffer (pH 8.0) for 15 minutes in a microwave. After washing with PBS containing 0.1% Tween-20 and treating with a 3% hydrogen peroxide/ethanol solution for 15 minutes at RT, tissues were blocked with PBS containing 5% bovine serum albumin for 30 minutes at RT, followed by incubation with first primary antibody overnight at 4℃. The next day, tissues were incubated with secondary antibodies for 30 min at RT, washed with PBS containing 0.1% Tween-20, and incubated with fluorophore solution (in a dilution of 1:100 in 1× Plus Manual Amplification Diluent) for 10 minutes at RT. Antigens were retrieved once again to remove non-specifically bound fluorophores, and the above steps were repeated until the last target protein completes the fluorophore labeling. 4’,6-diamidino-2-phenylindole (DAPI) were incubated for 5 minutes at RT for nuclei staining.

Following primary antibodies were used for immunofluorescence staining: anti-PTN (ABClonal, Cat#A3124, RRID: AB_2764921), anti-Collagen I/Col1A1 (abcam, Cat#ab34710, RRID: AB_731684), anti-CD44 (proteintech, Cat# 15675-1-AP, RRID: AB_2076198), and anti-Cytokeratin (Maxim, Cat#kit-0025, RRID: AB_3076318).

All images were captured and analyzed using an InForm Cell Analysis 2.2 (PerkinElmer). For analysis and quantification of expression level of PTN and Collagen I, cytoplasmic or membrane proteins were evaluated using the H-score with the following formula: H-score = 3× (% of strongly staining cells) + 2× (% of moderately staining cells) + 1 × (% of weakly staining cells), which gives a range of 0~300. To exploration the spatial distribution patterns of PTN and CD44, multi-layer images were analyzed using the APTime software developed by SODA Data Technology Inc. Briefly, we performed hierarchical segmentation of the tumor tissue region, starting from the tumor boundary and moving inward in 50 μm increments, based on tissue morphology to defined the boundaries of individual tumor lesions. The distribution density of $CD44^+$, PTN^+ and $CD44^+PTN^+$ tumor cells in each layer were then quantified.

Statistical analysis

An unpaired Student's t-test was used for comparisons between two groups, and one-way analysis of variance (ANOVA) was used for multiple comparisons with normal distribution. Linear regression was used for correlation analysis. Quantitative data are presented as the mean ± SEM. *P* values were annotated in the figures. All statistical analyses were conducted using GraphPad Prism 9.0.

1 Bagaev, A. *et al.* Conserved pan-cancer microenvironment subtypes predict response to immunotherapy. *Cancer Cell* **39** (2021).

2 Tang, F. *et al.* E3 ligase Trim35 inhibits LSD1 demethylase activity through K63-linked ubiquitination and enhances anti-tumor immunity in NSCLC. *Cell Rep* **42**, 113477 (2023).
